# Diffuse leukoencephalopathy in a 29-year-old male with hypertensive emergency

**DOI:** 10.1259/bjrcr.20150199

**Published:** 2016-11-02

**Authors:** Ghada Issa, Samer Nasser, Samir Kodsi, Zein Farhat

**Affiliations:** ^1^Department of Diagnostic Radiology and Nuclear Medicine, University of Maryland Medical Center, Baltimore, MD, USA; ^2^Department of Nephrology, Conemaugh Memorial Medical Center, Johnstown, PA, USA; ^3^Department of Diagnostic Radiology, Conemaugh Memorial Medical Center, Johnstown, PA, USA; ^4^Department of Health Services Research, University of Maryland, Baltimore, MD, USA

## Abstract

Severe hypertension is associated with multiple symptoms that reflect the end-organ damage effect of rapidly increasing blood pressure. Encephalopathy is a manifestation of the clinical spectrum of hypertensive emergencies. Hypertensive encephalopathy was initially described as part of the posterior reversible encephalopathy syndrome, which mostly involved the parieto-occipital white matter of the brain. A more detailed review of this syndrome reveals many cases where the brain abnormalities are distributed in a more random pattern. We describe a case of diffuse leukoencephalopthy in a young male who presented with altered mental status, ataxia, and blurred vision. This is the most diffuse brain involvement ever described in hypertensive statuses.

## Clinical presentation

A 29-year-old Caucasian male was sent to the local emergency room by his ophthalmologist after finding out that his blood pressure was 250/150 mmHg and he had retinal haemorrhages. The patient had a 2-week history of blurred vision, 2-month history of headache and a few days history of ambulatory dysfunction. In addition, the family members reported change in his mental status, as he seemed confused lately. The patient denied any chest pain, dyspnoea, arthralgias or urinary symptoms.

He was admitted to the medical service. A detailed history was taken. He had no significant past medical history. He admitted to heavy alcohol consumption previously but had cut down on his alcohol intake 2 weeks prior to presentation. He denied smoking and illicit drug use. He did not consume any over-the-counter drugs, except for occasional ibuprofen for his headaches. Particularly, he reported no ingestion of black liquorice, which excluded the liquorice-induced hypermineralocorticoid-like effect. A remarkable finding on examination was an S4 gallop. Physical features of Cushing disease, including central obesity and purple striae on the abdomen, were not present. His neurological examination was only significant for visual disturbance.

## Differential diagnosis

The differential diagnoses of hypertension and encephalopathy include primary and secondary hypertension, vasculitis, drug toxicity, eclampsia and pre-eclampsia in females, hepatic and uremic encephalopathy, pheochromocytoma, subarachnoid haemorrhage and acute stroke.

## Investigations/imaging findings

**Figure 1.**
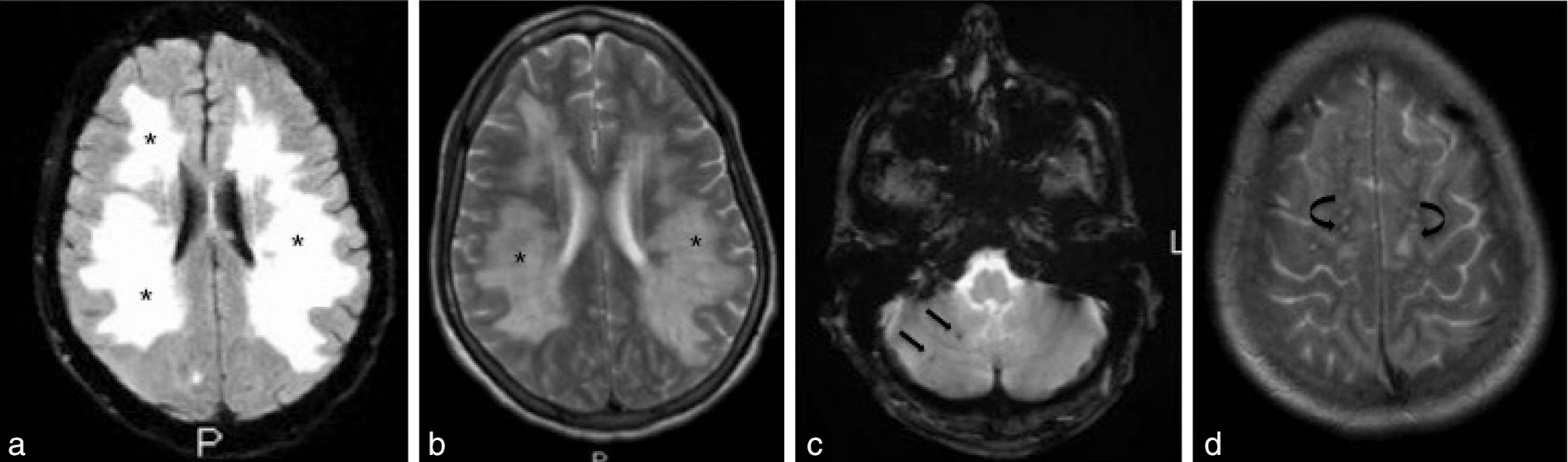
Axial fluid-attenuated inversion-recovery (a) and *T*_2_ (b) weighted images showing diffuse bihemispheric leukoencephalopathy and vasogenic oedema (asterisks). There are also two foci of petechial haemorrhage (straight arrows) in the right cerebellar hemisphere on axial gradient echo images (c), and subcortical white matter changes (curved arrows) in watershed distribution on axial *T*_2_ (d) weighted images.

**Figure 2.**
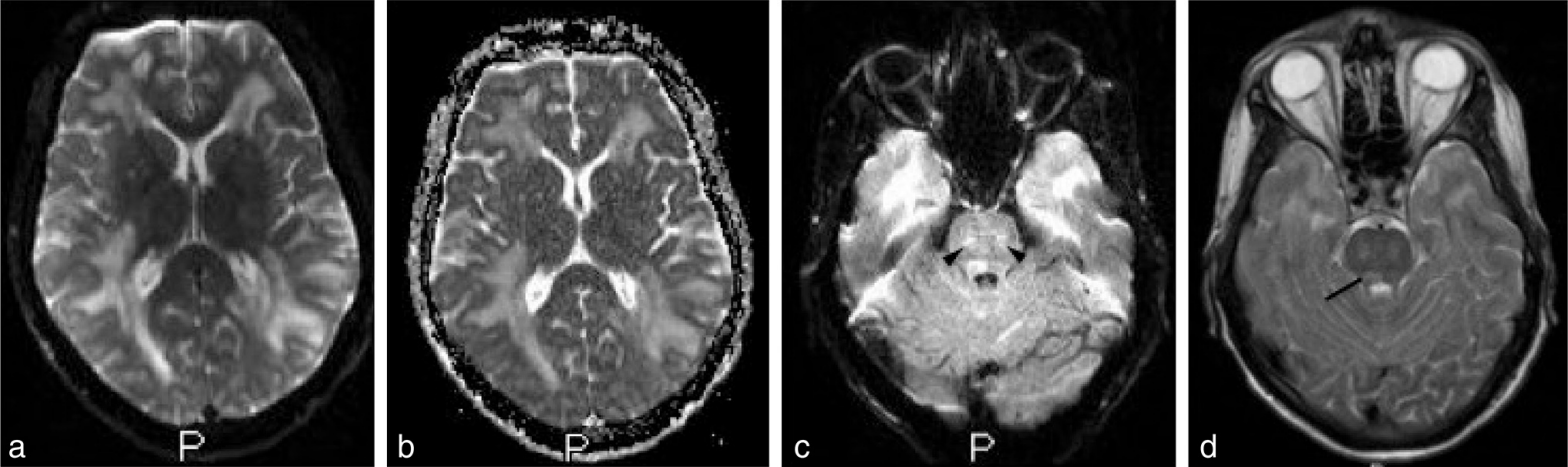
Axial diffusion weighted (a) and apparent diffusion coefficient (b) images with no evidence of restriction. Axial fluid-attenuated inversion-recovery (c) and *T*_2_ (d) weighted images showing the brain stem lesions with bilateral symmetrical involvement of the pons (arrowheads) and the right facial colliculus (arrow).

Diagnostic investigations revealed a creatinine level of 1.6 mg dl^−1^ and blood urea nitrogen of 18 mg dl^−1^. His sodium level was 139 meq l^−1^, potassium 3.2 meq l^−1^, bicarbonate 21 meq l^−1^ and chloride 109 meq l^−1^. His haemoglobin level dropped from 12.0 to 10.5 g dl^−1^ in 1 day. His indirect bilirubin level was elevated at 0.9 mg dl^−1^ (normal < 0.5 mg dl^−1^). His lactate dehydrogenase level was elevated at 352. Haptoglobin level was initially low. Peripheral smear, however, was negative for schistocytes. The next day, his lactate dehydrogenase level dropped and creatinine improved. The haemolysis was blamed on microangiopathy from the hypertensive crisis. An MRI of the brain was performed, which showed extensive bihemispheric supratentorial (Figure 1a,b,d) and infratentorial (Figure 2c,d) subcortical leukoencephalopathy involving portions of the splenium of the corpus callosum and pons but sparing the subcortical U-fibres. There were areas of petechial haemorrhage involving the cerebellum (Figure 1c). No evidence of restricted diffusion or post-contrast enhancement was seen (Figure 2a,b). The appearance was highly suggestive of acute hypertensive encephalopathy. MR angiography was unremarkable. A drug screen was performed on the patient and was negative. Serological evaluation (antinuclear antibodies, anti-neutrophil-cytoplasmic antibodies and rheumatoid factor) was negative and his complement fixation tests were normal. His aldosterone to renin ratio was < 20. His catecholamine levels were mildly elevated but the patient was on clonidine.

## Treatment

The patient was started on labetalol drip for control of his blood pressure. He was also started on oral clonidine and lisinopril. His blood pressure was better controlled over the course of 3 days.

## Outcome and follow-up

**Figure 3.**
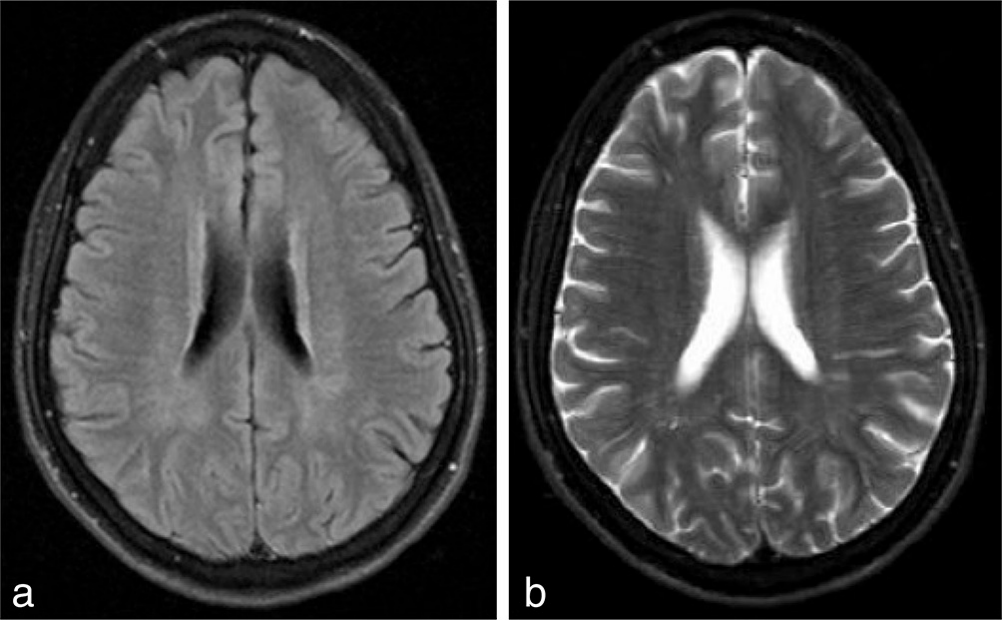
Axial fluid-attenuated inversion-recovery (a) and *T*_2_ weighted (b) images showing significant reversibility of the vasogenic oedema and subcortical white matter changes.

**Figure 4.**
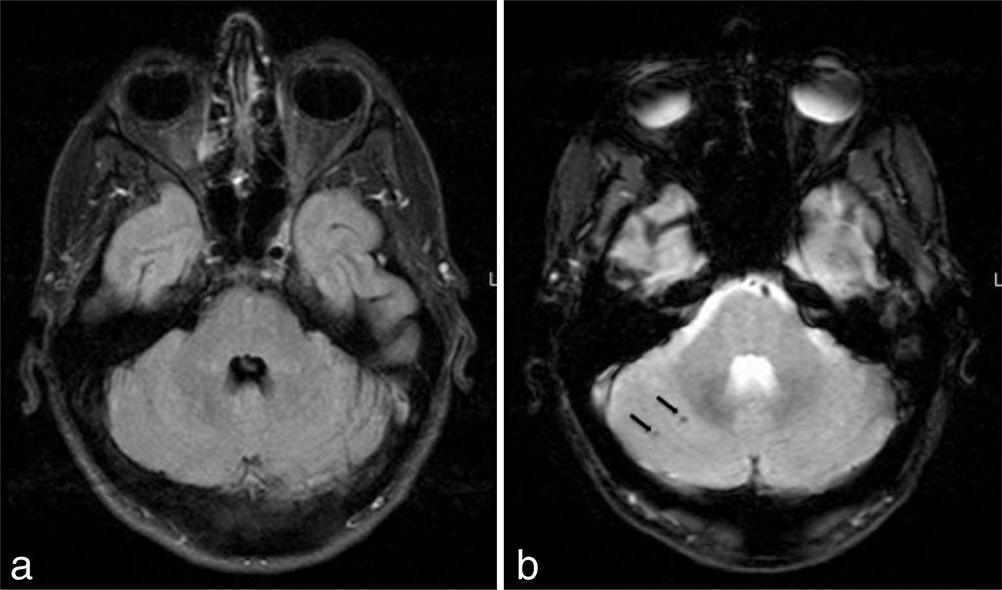
Resolution of the pontine lesions on axial fluid-attenuated inversion-recovery image (a). Persistence of the petechial haemorrhage (arrows) on gradient echo image (b).

On outpatient follow-up, the patient had better control of his blood pressure, ranging from 130–150 mmHg systolic and 70–92 mmHg diastolic. The patient was weaned off the clonidine. There was significant improvement in his vision and ophthalmological examination showed decrease in retinal haemorrhages. The 24-h urine collection for catecholamines was repeated and showed normalized values. Repeat MRI was performed after 1 month and showed complete resolution of the previous abnormalities with the exception of the petechial haemorrhage (Figures 3 and 4). He was advised to continue the current medications and a low-salt diet.

## Discussion

Reversible posterior leukoencephalophy syndrome is a clinicoradiological syndrome that was first described by Hinchey et al^1^ in 1996. It consists of acute neurological symptoms associated with reversible bilateral parieto-occipital white matter abnormalities on imaging, in the setting of hypertension.^1^ Since then, many reports have described similar clinicoradiological associations in different settings and with a wider spectrum of imaging findings that involved both the white and the grey matter. The name of the syndrome was changed to posterior reversible encephalopathy syndrome (PRES).^2^ However, the term is still controversial. The radiological findings can extend beyond the parieto-occipital regions, and sometimes even spare them. A recent study of the MRI patterns of PRES reported a higher rate of involvement of the frontal lobes; however, in all the cases, the extent of lesions was mostly limited to three regions of the brain and was never diffuse.^3^

The case described in this report did not have the classic distribution of radiological abnormalities associated with PRES. The two largest series conducted on patients with PRES included 120 and 50 subjects. Multilobar involvement was frequent in these series, but the most commonly affected lobes were the posterior (parieto-occipital) lobe in 94% and 65%, respectively, followed by the frontal lobe in 77% and 54%, respectively.^3,4^ Temporal, cerebellar and brain stem involvement was rare.^3^ Lesions were initially described as mostly subcortical but that was not supported by the findings of Kastrup et al,^3^ where the majority of cases had both cortical and subcortical involvement.^4^ In this case, the signal abnormalities were solely subcortical, more pronounced in the supratentorial region and overall involved six regions of the brain: frontal, temporal, parietal, occipital, cerebellar and brain stem. The most diffuse radiological findings reported in the literature involved five regions, and this was described in three PRES cases only.^3^

Atypical radiological features of this case also included involvement of the deep white matter and extension of the signal abnormalities to the ventricular surface.^5^ Not surprisingly, the association between these features and extreme hypertension has been described previously.^6^ Our report supports this association, where the recorded blood pressure of the patient was 250/150 mmHg, which is much higher than the mean blood pressure of 191/104 mmHg seen in PRES.^4^ Symmetry is not a significant finding, being present in one-half of the cases in most reports.^2–4^

Another important finding is the presence or absence of restricted diffusion. This finding reflects the underlying pathophysiology of PRES, which is still being debated. The absence of restricted diffusion, as in this patient’s MRI, supports the theory of hyperperfusion and vasogenic oedema. It was postulated that acute hypertension can result in increased permeability of the blood–brain barrier and secondary extravasation of fluids and proteins, resulting in vasogenic oedema.^7^ The predilection of the posterior region of the brain in PRES was thought to be related to its poor sympathetic innervation; therefore, the cerebral vessels are less likely to vasoconstrict in response to hypertension and are more prone to breakdown.^4^ However, as more reversible encephalopathy syndromes were described outside the posterior regions, a second theory of hypoperfusion and cytotoxic oedema was then proposed. It implicated that autoregulatory vasoconstriction in response to hypertension can be so pronounced in some areas of the brain that it causes ischaemia and subsequently cytotoxic oedema and restricted diffusion on MRI sequences.^2–4^ In addition, there is a third alternative theory that also supports the hypoperfusion state in PRES. According to this theory, vasoconstriction is caused by endothelial dysfunction, which is, in turn, the result of the immunotoxic effects of vasoactive agents released in response to increased intracranial pressure from hypertension.^6^

In most cases, PRES is reversible, and a worse prognosis was only associated with the presence of haemorrhage on imaging, commonly of the microhaemorrhage type.^5^ Our patient did have petechial haemorrhage in the cerebellum, but he had a benign outcome, with complete resolution of the clinical symptoms after 1 month. The persistence of petechial haemorrhage on follow-up MRI suggests that this finding might be of a chronic nature. No relationship has yet been found between the extent of oedema and the severity of hypertension.^5^

PRES can occur in multiple settings. The most common aetiology remains hypertension. In this case, it falls under the spectrum of hypertensive emergencies that is defined as symptomatic hypertension with blood pressure > 180/120 mmHg and end-organ involvement.^8^ Other conditions include cytotoxic medications, infection and sepsis, autoimmune diseases, toxaemia of pregnancy, renal disease, severe hypercalcaemia and hypomagnesaemia.^2–5^ The only statistically significant associations between the aetiology of PRES and the location of radiological abnormalities were the involvement of the cerebellum in autoimmune disease and cortical involvement in infection states.^4^ An extensive work-up was conducted for the patient presented in this report. The only identified pathology was hypertension, and no underlying aetiology of the hypertension was found. Hypertensive encephalopathy most commonly affects middle-aged individuals with chronic hypertension and is slightly more prevalent in men. It is frequently encountered in black people, following the higher prevalence of hypertension in this ethnic group.^8^ Altogether, the case of this 29 year-old previously healthy Caucasian male did not reflect the known features of PRES, and had a specially unique radiological presentation.

## Learning points

1. Diffuse brain involvement with vasogenic oedema should be included in the wide spectrum of radiological findings associated with reversible hypertensive encephalopathy.
2. The extent of imaging abnormalities is not related to the severity of hypertension.
3. A thorough diagnostic work-up is needed to identify any underlying aetiology of encephalopathy.
4. Clinicians should suspect hypertensive encephalopathy in every setting where there are neurological symptoms and brain abnormalities on imaging, regardless of the distribution.

## Consent

Written informed consent was obtained from the patient for publication of this case report, including accompanying images.
